# Publisher Correction: Dissociable roles of cortical excitation-inhibition balance during patch-leaving versus value-guided decisions

**DOI:** 10.1038/s41467-021-23485-2

**Published:** 2021-06-17

**Authors:** Luca F. Kaiser, Theo O. J. Gruendler, Oliver Speck, Lennart Luettgau, Gerhard Jocham

**Affiliations:** 1 Biological Psychology of Decision Making, Institute of Experimental Psychology, Heinrich Heine University, Düsseldorf, Germany; 2 Center for Behavioral Brain Sciences, Otto von Guericke University, Magdeburg, Germany; 3 Center for Military Mental Health, Military Hospital Berlin, Berlin, Germany; 4 Leibniz Institute for Neurobiology, Magdeburg, Germany; 5 German Center for Neurodegenerative Diseases (DZNE), Magdeburg, Germany; 6 Department of Biomedical Magnetic Resonance, Institute for Physics, Otto von Guericke University, Magdeburg, Germany

**Keywords:** Cognitive neuroscience, Reward, Human behaviour

Correction to: *Nature Communications* 10.1038/S41467-020-20875-W, published online 10 February 2021.

The original version of this Article contained an error in the “Methods” section, under the “Decision-making task” heading. The results of a *t* test were reported with the incorrect degrees of freedom, incorrectly written as “t18858 = −20.915, *p* < 0.001.” The correct version replaced “t18858” with “t18558.” This has been corrected in both the PDF and HTML versions of the Article.

The original version of this Article also contained an error in the “Methods” section titled “Behavioural modelling of value-guided decisions” in which Eqs. 5 and 6 inadvertently swapped during production. The incorrect version stated

Since it is known that humans do not weigh magnitudes and probabilities in a statistically optimal way, we considered systematic distortions in the weighting of reward information in our models (*u*(*m*) and *w*(*p*), for reward magnitudes and probabilities, Eqs. 5 and 6, respectively)^56^.

5 $$u(m_{\rm{O}})={m_{\rm{O}}}^{\alpha }$$

where *p*_O_ are the objective reward probabilities and *γ* is a free parameter used to fit subjective reward probabilities. Subjective magnitudes were estimated by:

6 $$w(p_{\mathrm{O}})={\frac {{p_{\mathrm{O}}}^{\gamma }}{\left({p_{\mathrm{O}}}^{\gamma }+(1-{p_{\mathrm{O}}})^{\gamma }\right)^{1/\gamma }}}$$

The correct version states

Since it is known that humans do not weigh magnitudes and probabilities in a statistically optimal way, we considered systematic distortions in the weighting of reward information in our models (*w*(*p*_O_) and *u*(*m*_O_) for reward probabilities and magnitudes, Eqs. 5 and 6, respectively)^56^.

5 $$w(p_{\mathrm{O}})={\frac {{p_{\mathrm{O}}}^{\gamma }}{\left({p_{\mathrm{O}}}^{\gamma }+(1-{p_{\mathrm{O}}})^{\gamma }\right)^{1/\gamma }}}$$

where *p*_O_ are the objective reward probabilities and *γ* is a free parameter used to fit subjective reward probabilities. Subjective magnitudes were estimated by:

6 $$u(m_{\rm{O}})={m_{\rm{O}}}^\alpha$$

The original version of this Article also contained an error in the Methods section under the heading “MRS data acquisition”, in which the terms “rM1 and lM1” were incorrectly ordered. The incorrect version stated

Average M1 voxel centroids in standard space were estimated at MNI *x* = −28.97 ± 0.82, *y* = −18.48 ± 0.92, *z* = 51.86 ± 0.59 and MNI *x* = 31.90 ± 0.71, *y* = −14.76 ± 1.06, *z* = 49.76 ± 0.88 for rM1 and lM1, respectively.

The correct version states

Average M1 voxel centroids in standard space were estimated at MNI *x* = −28.97 ± 0.82, *y* = −18.48 ± 0.92, *z* = 51.86 ± 0.59 and MNI *x* = 31.90 ± 0.71, *y* = −14.76 ± 1.06, *z* = 49.76 ± 0.88 for lM1 and rM1, respectively.

