# Supplementary material for: Dissociable roles of cortical excitation-inhibition balance during patch-leaving versus value-guided decisions
Source: Nat Commun. 2021 Feb 10;12:904. doi: 10.1038/s41467-020-20875-w (PMC7875994; doi:10.1038/s41467-020-20875-w)
Supplement: Supplementary file 1 — Supplementary Information [file 41467_2020_20875_MOESM1_ESM.pdf]

Dissociable roles of cortical excitation-inhibition balance during patch-leaving versus value-guided decisions

Supplementary Information

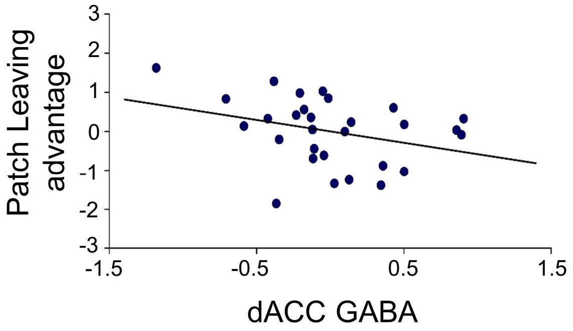

Supplementary Figure S1. Relationship between dACC GABA and patch-leaving. GABA contribution to the effect of E/I balance on patch-leaving shown in main figure 2C. Higher dACC concentrations of GABA are, by trend, associated with earlier patch leaving (lower average patch leaving advantages) (Pearson correlation on residuals (compare main text and methods): r = -0.323, p = 0.087, CI_{95} = [-0.617 – 0.049]; N = 29). Source data are provided as a Source Data file.

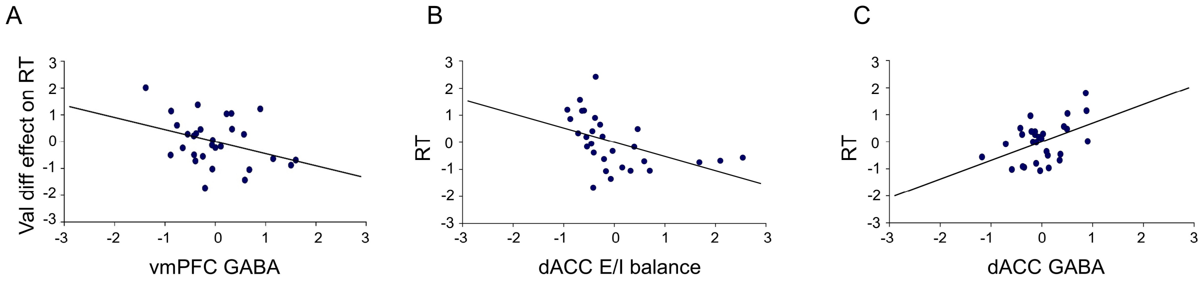

Supplementary Figure S2. Additional analysis for the relationship between value-guided choice and cortical neurochemistry. A) GABA contribution to the effect of E/I balance shown in main figure 3D. Participants' responses slowed down on difficult trials (trials with low value difference). This effect was related to vmPFC GABA concentrations (Pearson correlation on residuals: r = -0.357, p = 0.057, CI_{95} = [-0.640 – 0.011]). Val diff = value difference. B) dACC E/I balance relates to overall response speed during value guided choice (Pearson correlation on residuals: r = -0.459, p = 0.012, CI_{95} = [-0.707 – -0.111]). RT = Reaction Time. C) dACC GABA relates to overall response speed during value guided choice (Pearson correlation on residuals: r = 0.452, p = 0.014, CI_{95} = [0.102 – 0.702]). RT = Reaction Time. N = 29 in all figures. Source data are provided as a Source Data file.

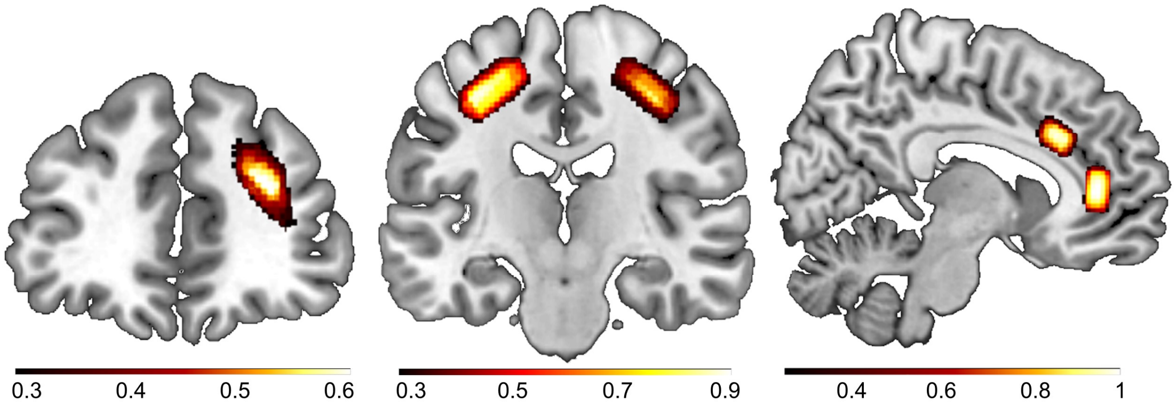

Supplementary Figure S3. Overlay of voxel placements across all participants: Average locations of all regions of interest. Brighter colors indicate a greater overlap across participants. Left: Average placement of dlPFC voxel. Middle: Average location of M1 voxels. Right: Average location of vmPFC and dACC MRS voxel. $N = 29$.

Supplementary Notes

1) Reaction times in the patch leaving phase are not influenced by trial-wise patch leaving advantages

We reran a regression model to analyze reaction times during patch-leaving decisions. Here, we included trial-wise patch leaving advantages instead of using costs and patch value differences as separate regressors. This analysis revealed no significant influence of PLA (t_{28} = -0.118, p = 0.907, CI_{95} = [-0.042 – 0.038], $U3_1$ = 0.483). We again find a significant influence of whether each trial was a switch trial or not (t_{28} = 3.776, p = 0.001, CI_{95} = [0.042 – 0.141], $U3_1$ = 0.310), of trial number (t_{28} = -8.039, p < 0.001, CI_{95} = [-0.310 – -0.184], $U3_1$ = 0.897), of whether the presentation side of patch values changed with respect to the last trial (t_{28} = 3.844, p = 0.001, CI_{95} = [0.025 – 0.083], $U3_1$ = 0.276) and of whether the value-guided choice in the last trial had been rewarded (t_{28} = 3.689, p = 0.001, CI_{95} = [0.020 – 0.071], $U3_1$ = 0.207). The finding that costs did influence reaction times whereas neither PLA nor patch value differences (analysis in main text) had an effect is likely related to the structure of the task. The cost of leaving is displayed on screen at the outset of each trial, whereas patch values have to be held in memory from the outcome of the last trial's patch choice.

2) vmPFC E/I balance relates to weighting of reward information during value-guided choice

In the main text, we report a negative relationship of vmPFC E/I balance and choice accuracy. To further investigate this, we wanted to quantify the degree to which participants' choices were guided by the options' expected values. To this end, we used a logistic regression which is already reported in the main text. Similar to our relationship between E/I balance and choice accuracy reported in the main manuscript, we found that E/I balance in vmPFC was related to the degree to which participants' choices were governed by expected values. There was a significant negative relationship between vmPFC E/I balance and the effect of value difference on choice (t_{22} = -2.593, p = 0.017, CI_{95} = [-0.959 – 0.107]; r = -0.484, p = 0.008, CI_{95}= [-0.722 – -0.143]). Thus, mirroring the effects on % correct choices, participants with higher levels of GABA relative to glutamate in vmPFC based their choices more strongly on the options' expected values. We further detailed this effect by re-running the regression with separate regressors for the differences in reward probabilities and magnitudes instead of one coding for difference in expected value. Participants used both reward probabilities (t_{28} = 11.708, p < 0.001, CI_{95} = [3.560 – 5.070], $U3_1$ = 0) and magnitudes (t_{28} = 14.431, p <0.001, CI_{95} = [2.806 – 3.734], $U3_1$ = 0) to guide their choices. Choices of participants with increased vmPFC E/I balance were more strongly influenced by reward probabilities compared to magnitudes (t_{22} = 2.736, p = 0.012, CI_{95} = [0.134 – 0.971]; r = 0.504, p = 0.005, CI_{95}= [0.169 – 0.735]). This pattern of results matches our findings reported in the main text and again indicates that participants with a greater E/I balance in vmPFC based their decisions less on objective differences in expected values. This effect is potentially mediated by a stronger reliance on reward probabilities than magnitudes.

3) Simultaneous regression of all behavioural parameters of interest against E/I balance in dACC and vmPFC

Some of our dependent variables may be correlated with each other across participants. This is expected since some of the tests investigate parameters that we assume to be driven by a

shared underlying mechanism[1]. For instance, consider the case for % correct choices on one hand and the effect of value difference on RT on the other. As can be seen from Supplementary Table 2, there is a negative correlation between these two variables, indicating that the (negative) effect of value difference on RT is most pronounced in participants with high percentage of correct choices. This, however, is exactly what would be mechanistically predicted from models using competition via mutual inhibition: Slowing the decision in the face of a lot of noise (a difficult trial with low value difference) allows for the choice to be dominated by the available evidence, while averaging out (neural) noise over time. To assess the orthogonal contributions of all the different behavioural parameters across both the patch-leaving and value-guided choice phase, we therefore included all of the parameters of interest from both phases (Supplementary Table 1 and 2) into one single regression model and now used either dACC or vmPFC E/I balance as the dependent variable. We still find a significant effect of patch leaving advantage on dACC E/I balance (t_{20} = 3.013, p = 0.007, CI_{95} = [0.175 – 0.961]) but no significant effect of any other variable of interest (all p > 0.119). When regressing the same design matrix against vmPFC E/I balance, we find no significant effect of any behavioural parameter (all p > 0.151).

4) Model Validation: Simulate and Recover

To validate our model fitting routines [2,3], we generated and recovered data for the model with the lowest BIC (prospect model with α and γ as free parameters). We generated 500 artificial data sets by randomly selecting α and γ parameters in the range between 0 and 3. We then recovered these parameters from the artificial data with the same procedure as used for our real participants. We used 1000 random starting points to find the combination of free parameters yielding the minimal negative log likelihood across iterations. All fittings were done for each participant separately. The distance and correlations between recovered parameters and the ground truth parameters across subjects were estimated (Supplementary Figure S4) as well as the correlations between recovered parameters.

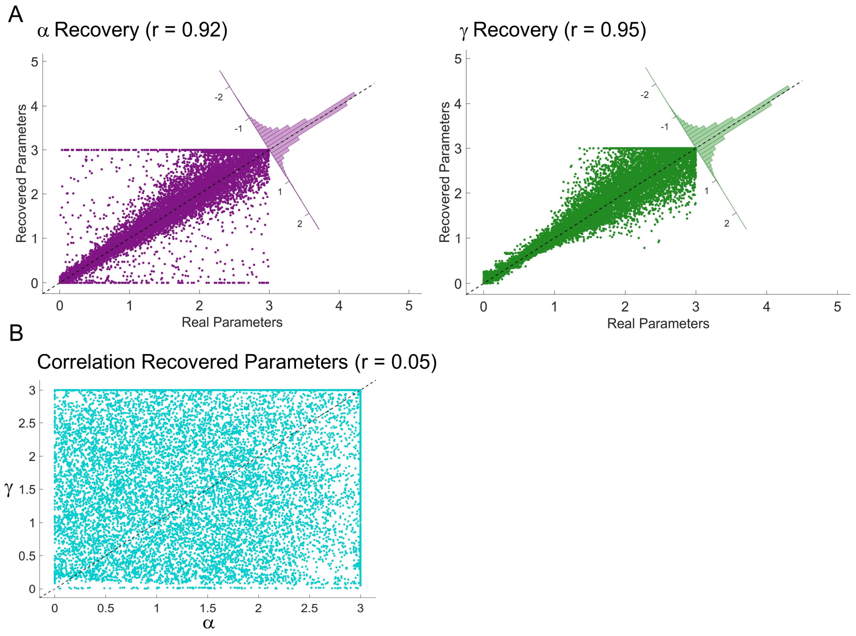

Supplementary Figure S4. Overview of Simulated and Recovered Model Parameters: A) Correlation between true and recovered parameters and a histogram of the difference between true and recovered parameters for our winning model (see methods for model details). B) Correlations between recovered parameters. Source data are provided as a Source Data file.

Supplementary Table 1: **Correlations between behavioral variables of interest during the patch–leaving phase.** Correlations > 0.4 are marked in red. PLA = Patch Leaving Advantage, RT = Reaction Time, Costs on RT = β regression weight of costs on reaction times. Source data are provided as a Source Data file.

	PLA	RT	Cost effect on RT
PLA	1	-0.08	0.13
RT	-0.08	1	-0.35
Cost effect on RT	0.13	-0.35	1

Supplementary Table 2: **Correlations between behavioral variables of interest during the value-guided choice phase.** Correlations > 0.4 are marked in red. RT = Reaction Time, val diff RT = β regression weight of value difference on RT, $\alpha =$ parameter transforming objective to subjective magnitudes, $\gamma =$ parameter transforming objective probabilities to subjective probabilities. Source data are provided as a Source Data file.

	% correct	RT	val diff RT	α	γ
% correct	1	0.42	-0.65	0.87	-0.64
RT	0.42	1	-0.09	0.49	-0.03
val diff RT	-0.65	-0.09	1	-0.63	0.36
α	0.87	0.49	-0.63	1	-0.38
γ	-0.64	-0.03	0.36	-0.38	1

Supplementary Table 3: **Correlation of E/I balance across cortical areas.** Overview of correlations between E/I balances between all regions of interest. Correlations > 0.4 are marked in red. dlPFC = dorsolateral prefrontal cortex, vmPFC = ventromedial prefrontal cortex, dACC = dorsal anterior cingulate cortex. Source data are provided as a Source Data file.

	dlPFC	M1 left	M1 right	vmPFC	dACC
dlPFC	1	0.15	0.22	-0.09	0.11
M1 left	0.15	1	0.18	-0.09	-0.22
M1 right	0.22	0.18	1	-0.19	0.04
vmPFC	-0.09	-0.09	-0.19	1	-0.38
dACC	0.11	-0.22	0.04	-0.38	1

Supplementary Table 4: Overview of neurochemical effects in all regression models. Neurochemical effects for all regression models conducted to analyze patch-leaving behavior. All regression models include all E/I balances (and an intercept) as dependent variables and the behavioural variable of interest as independent variable. *T*- and *p*- values indicate the test statistic for each coefficient in the regression model to test the null hypothesis that the coefficient is zero. Significant effects (p <= 0.05) are marked in red. Source data are provided as a Source Data file.

	dlPFC E/I [t-stat, p-value]	M1 left E/I [t-stat, p-value]	M1 right E/I [t-stat, p-value]	vmPFC [t-stat, p-value]	dACC [t-stat, p-value]
PLA	-0.39, 0.70	0.82, 0.42	1.32, 0.20	-0.20, 0.85	2.64, 0.02
RT	0.15, 0.88	0.87, 0.39	0.00,1	-0.16, 0.87	-1.33, 0.20
Effects of Cost on RT	-0.53,0.60	1.00, 0.33	0.57, 0.57	0.46, 0.65	2.19, 0.04

Supplementary Table 5: Overview of neurochemical effects in all regression models: Neurochemical effects for all regression models conducted to analyze value-guided choice behavior. All regression models include all E/I balances (and an intercept) as dependent variables and the behavioural variable of interest as independent variable. *T*- and *p*- values indicate the test statistic for each coefficient in the regression model to test the null hypothesis that the coefficient is zero. Significant effects (p <= 0.05) are marked in red. Source data are provided as a Source Data file.

	dlPFC E/I [t-stat, p-value]	M1 left E/I [t-stat, p-value]	M1 right E/I [t-stat, p-value]	vmPFC [t-stat, p-value]	dACC [t-stat, p-value]
% correct	-0.44, 0.67	-0.62, 0.54	-0.53, 0.60	-2.44, 0.02	-0.85, 0.41
RT	-0.24, 0.81	0.22, 0.83	-0.10, 0.92	-1.58, 0.13	-2.42, 0.02
Effect of Val Diff on RT	-0.41, 0.68	1.68, 0.11	0.53, 0.60	2.88, 0.01	1.28, 0.21
α	-0.29, 0.77	-0.55, 0.59	-0.47, 0.64	-2.41, 0.02	-1.14, 0.27
γ	0.49, 0.63	1.00, 0.33	0.14, 0.89	2.14, 0.04	0.02, 0.98

Supplementary Table 6: Parameter values and model fits for behavioural models. Overview of all recovered model parameters as well as their model fit (BIC = Bayesian Information Criterion). The model with the lowest BIC is bold. See Supplementary Analysis 4 for model validation. EV models assume no distortions in value weighting. EU models assume distortions in reward magnitude weighting, EVPW in reward probabilities and SU in both reward probabilities and magnitudes. Additive (Add) models assume additive value integration, multiplicative (multi) models assume multiplicative value integration, and hybrid models a combination of both. Source data are provided as a Source Data file.

**Model**	$\frac{\omega_{mult}}{\omega_m+\omega_p+\omega_{mult}}$	$\frac{\omega_m}{\omega_m+\omega_p}$	$\frac{\omega_p}{\omega_m+\omega_p}$	ω_{mult}	α	γ	BIC
**EV**							
Add		0.08 ± 0.01	0.92 ± 0.01				182.78 ± 6.47
Multi				1.93 ± 0.18			223.44 ± 11.75
Hybrid	0.27 ± 0.05	0.17 ± 0.06	0.83 ± 0.06				166.45 ± 7.16
**EU**							
Add		0.66 ± 0.06	0.34 ± 0.06		0.22 ± 0.06		174.23 ± 6.92
Multi				5.54 ± 0.73	0.71 ± 0.07		164.40 ± 7.92
Hybrid	0.54 ± 0.06	0.65 ± 0.08	0.35 ± 0.08		0.59 ± 0.08		165.61 ± 7.51
Fix$_{\omega_{mult}}$ Multi					0.69 ± 0.06		172.06 ± 7.91
**EVPW**							
Add		0.08 ± 0.01	0.92 ± 0.01			1.03 ± 0.04	187.46 ± 6.46
Multi				3.11 ± 0.27		1.79 ± 0.15	180.78 ± 8.49
Hybrid	0.38 ± 0.07	0.23 ± 0.06	0.77 ± 0.06			1.23 ± 0.09	168.51 ± 7.21
Fix$_{\omega_{mult}}$ Multi						1.84 ± 0.15	182.23 ± 8.41
**SU**							
Adi		0.65 ± 0.06	0.35 ± 0.06		0.23 ± 0.06	1.03 ± 0.04	178.88 ± 6.89
Multi				10.04 ± 1.80	0.59 ± 0.06	0.83 ± 0.07	162.96 ± 7.78
Hybrid	0.58 ± 0.06	0.64 ± 0.08	0.36 ± 0.08		0.65 ± 0.08	1.14 ± 0.07	168.99 ± 7.50
Fix$_{\omega_{mult}}$ Multi					0.58 ± 0.05	0.88 ± 0.05	**161.99 ± 7.06**

Note: All values are mean values across participants ± standard error of the mean. N = 29.

Supplementary Note: Exploratory Findings - Relating current findings to own previous work

In an earlier study, we have already reported relationships between vmPFC E/I balance and optimal choice behaviour[4]. In particular, we had reported that high levels of GABA, and low levels of glutamate, respectively, were related to participants' performance on difficult trials (those with low value difference), as measured by the softmax inverse temperature. This finding is exactly predicted by mechanistic models based on competition by mutual inhibition[4]. However, a recent study found that choices were more strongly guided by multiplicative as opposed to additive value computation after administration of the NMDA receptor agonist d-cycloserine to healthy volunteers[5]. Combining values multiplicatively is considered more optimal whereas an additive value integration is potentially less complex. In our own previous data, we found an effect of vmPFC E/I balance on softmax inverse temperature[4]. In this work, however, we had not compared between different models featuring multiplicative versus additive value construction, or a mixture of both. We have therefore reanalyzed our previous data with the same set of models as used in the current study. All magnitudes have been rescaled between 1 and 10 prior to model fitting. We find that a hybrid model with no distortions in value weighting fits the data best. Since the EV hybrid model fits our previous data best, we assessed the relationship between this model's free parameters and E/I balance. One participant had to be excluded because GABA and glutamate could not be successfully detected[4]. We don't find any significant relationship between vmPFC GABA (t_{21} = -1.559, p = 0.134) or glutamate (t_{21} = 0.421, p = 0.678) on the reliance of integrative versus additive value integration. When we compared the reliance on multiplicative versus additive value updating in our current data set (EV hybrid), we find, as expected, a greater reliance on multiplicative value integration with a lower vmPFC E/I balance (t_{22} = -2.423, p = 0.024) as well as a greater reliance on magnitude compared to probability values within the additive module (t_{22} = -2.711, p = 0.013). Neither the previous nor this study was primarily designed to study whether E/I balance measured with MRS relates to a multiplicative or additive value integration. It would be interesting for further

studies to analyze this question with a set of options where choices would explicitly dissociate multiplicative from additive value integration.

As reported in the main text, for our present study, we find that a multiplicative SU model fits the data best. However, we did not obtain sufficient model recovery for the choice stochasticity parameter and therefore decided to fix it at the median recovered vale. There are a number of possible reasons for this. First, in the 2012 data, the trials' combination of reward attributes had been specifically optimized (offline) for the value-guided choice task to allow a certain level of difficulty, to control for correlation between chosen and unchosen value, and to incorporate a certain range of no-brainer trials. In contrast, in the current task, reward magnitudes are generated from the chosen patch, a random fraction of which is allocated to the two patches. Small magnitude differences are therefore less likely to occur, which potentially prevents a reliable estimation of the choice stochasticity parameter. Secondly, in the current task the distortion of reward magnitudes becomes more important since magnitudes can potentially cover a wider range of values that depends on the current patch value, as opposed to a fixed minimum and maximum in the 2012 study.

Supplementary Table 7: Overview of model fitting to the data presented in Jocham et al. (2012)[4]. EV models assume no distortions in value weighting. EU models in reward magnitude weighting, EVPW in reward probabilities and SU in reward probabilities and magnitudes. Add models assume additive value integration, multi models multiplicative value integration and hybrid models a combination of both. Source data are provided as a Source Data file.

**Model**	$\frac{\omega_{mult}}{\omega_m+\omega_p+\omega_{mult}}$	$\frac{\omega_m}{\omega_m+\omega_p}$	$\frac{\omega_p}{\omega_m+\omega_p}$	ω_{mult}	α	γ	BIC
**EV**							
Add		0.07 ± 0.00	0.93 ± 0.00				237.05 ± 8.16
Multi				1.80 ± 0.17			257.36 ± 15.11
Hybrid	0.19 ± 0.04	0.10 ± 0.04	0.90 ± 0.04				**224.69 ± 9.56**
**EU**							
Add		0.44 ± 0.06	0.56 ± 0.06		0.40 ± 0.06		229.43 ± 8.50
Multi				3.70 ± 0.47	0.77 ± 0.06		231.97 ± 10.46
Hybrid	0.27 ± 0.05	0.37 ± 0.08	0.63 ± 0.08		0.69 ± 0.06		227.20 ± 9.43
**EVPW**							
Add		0.07 ± 0.00	0.93 ± 0.00			1.02 ± 0.04	241.89 ± 8.02
Multi				2.07 ± 0.15		1.47 ± 0.16	243.44 ± 11.62
Hybrid	0.22 ± 0.04	0.06 ± 0.01	0.94 ± 0.01			1.09 ± 0.08	228.48 ± 9.43
**SU**							
Add		0.44 ± 0.06	0.56 ± 0.06		0.40 ± 0.06	1.00 ± 0.04	234.20 ± 8.30
Multi				8.74 ± 1.06	0.55 ± 0.04	0.67 ± 0.03	228.09 ± 9.38
Hybrid	0.29 ± 0.06	0.35 ± 0.08	0.65 ± 0.08		0.70 ± 0.05	1.03 ± 0.06	231.60 ± 9.29

Note: All values are mean values across participants ± standard error of the mean. *N* = 25.

Supplementary Note: Exploratory Analysis - Drift Diffusion Modelling of Choice Data

To obtain a formal characterization of the process of evidence accumulation across trials, we fitted a hierarchical drift diffusion model (DDM)[6]. In brief, DDM assume that choices between two alternatives depend upon accumulation of noisy evidence until a decision threshold is reached. The model thereby not only predicts choice probabilities but also response time (RT) distributions. The predicted choice probabilities and RTs critically depend upon three free parameters. First, the decision boundary *a* determines how much evidence needs to be accumulated. Second, the drift rate *v* captures the speed at which the evidence accumulation process approaches either boundary[6]. Third, RT is assumed not to solely depend on the choice process itself, but also on other non-decisional processes like stimulus perception and the execution of a motor response, which is reflected in the non-decision time (*ndT*)[7]. To account for across-trial variations[8], we also tested the effects of variability in *ndt* (*st*) and *v (sv*).

We used the Bayesian hierarchical drift diffusion modeling toolbox with default priors[6] in Python 2 to infer latent variables underlying response time distributions of patch leaving trials and correct vs. incorrect value - guided choices. The estimation of individual parameters is hierarchical since they are not assumed to be independent of one another but drawn from an underlying group distribution[6]. We estimated drift rate, boundary separation and non-decision time individually, but across-trial variability in drift rate and non-decision time on a group level[9,10]. For model comparisons, we included additional effects of bias towards one decision boundary (*z*) and variations in bias (*sz*) in patch leaving trials as well as linear regression models assessing the effect of reward information onto free DDM parameters. For regressions, all continuous variables were z-scored per participant before estimating regression coefficients. Cost levels were z-scored on a group level. Since our task does not involve a maximum response time, we excluded all trials with response times below 0.3 or above 4 seconds before model fitting. Additionally, we specified 5 % of responses to be contaminants. The toolbox uses Markov-Chain Monte Carlo sampling for a Bayesian approximation of the posterior distribution of each model parameter. For every model, we ran

thirty separate Markov chains and report parameter estimates and posterior distributions of a concatenated model across all chains[9]. We generated 5000 samples for every chain and discarded one half of all samples as burn-in[9]. Every third sample was discarded for thinning, thereby reducing autocorrelations in the chains. To assess model convergence, we inspected the sampled posterior traces, their autocorrelation and the Gelman-Rubin $\hat{R}$ statistics, which compares between and within chain variance[6,11]. $\hat{R}$ for a group level parameter with a distance of > 0.02 from one were defined as non-converged models. To compare between models, we used the Deviance Information Criterion (DIC) where a lower DIC points towards a better fit. Based on previous findings[12–16], we predicted a relationship between E/I balance and the drift rate *v* and decision boundary *a*.

For the patch leaving phase we find that a drift diffusion model with *a, v, ndt*, *z*, *st* and *sz* fit the data best. When we assess the effects for individual model parameters for their relationship with E/I balance, we find a significant effect of dACC E/I balance on drift rate ($t_{23} = 2.011$, p = 0.056, CI_{95} = [-0.012 – 0.837], $r = 0.387$, $p = 0.038$, CI_{95}= [0.023 – 0.660]). This indicates that participants with a greater dACC E/I balance show a higher drift towards stay decisions and confirms our model free findings. There were no significant effects in any other region of interest (all $p > 0.597$) nor with decision boundary (all $p > 0.269$). We included E/I balances directly in the model rather than correlating E/I balances with individual slopes (after model fitting) since the latter might be biased towards the group mean. However, since the standard DDM without incorporating E/I balance fitted the patch-leaving data best, we ran the exploratory analysis reported above.

For the value guided choice phase, we find that a DDM incorporating a regression model on drift rate fits the accuracy-coded data best. While we do not find any main effect of vmPFC E/I on drift rate (highest posterior density interval (HPDI): [-0.279 – 0.052]), we find an interaction effect between vmPFC E/I and the effects of value difference on drift rate (HPDI: [0.032 – 0.124], <0.001 % of the posterior distribution below zero). Additionally, we find an overall greater drift rate with higher value difference between options (HPDI: [0.515 – 0.627], <0.001% of distribution below zero) and in no brainer trials (HPDI: [1.843 – 2.117],

<0.001% of distribution below zero). This pattern of results matches our findings obtained in regression analyses and again points towards an influence of vmPFC E/I balance onto the speed of value integration.

Supplementary Table 8: Overview of DDM models: Overview of HDDM model specifications.

**Free Parameters**	**Linear Model**	**DIC**	**Gelman - Rubin**
Patch Leaving Phase			
a, v, ndt, sv, st		3145.12	yes
a, v, ndt, z, sz, st		3104.46	yes
a, v, ndt, z, st, sz	*v*~1+costs+dacc+costs:dacc	4205.75	yes
a, v, ndt, z, st, sz	*a*~1+costs+dacc+costs:dacc	4189.73	no
Value guided choice phase			
a, v, ndt, sv, st		15112.98	yes
a, v, ndt, sv, st	*v*~1+valdiff+NB+vmpfc+valdiff:vmpfc	11818.15	yes
a, v, ndt, sv, st	*a*~1+valdiff+NB+vmpfc+valdiff:vmpfc	14290.62	no

Supplementary Methods

Details of the behavioural task

All stimuli were presented on a grey (RGB: 60, 60, 60) background with a contrast optimized for the MEG recording chamber on a screen in a distance of one meter from the sitting participants. Stimuli were displayed via a projector with a refresh rate of 75 Hz located outside the MEG recording chamber. During patch-leaving, participants were presented with two patches (RGB: 80, 80, 80) framed with a white outline indicating in which patch participants are currently staying. If participants chose to switch they had to pay a travelling cost indicated by the size of a grey bar (RGB: 160, 160, 160) presented between both

patches. In trials where participants chose to switch, the rectangular bar signaling switch costs turned red (RGB: 178, 70 70) and the respective costs were subtracted from the subjects total earnings up to this trial. Afterwards the current patch values were revealed. Patch Values were presented in blue (RGB: 69,102,174). In both stages of the experiment a blue progress bar (RGB: 65,105, 204) was shown at the bottom of the screen indicating subjects current score. Participants selected an option by means of a button press with the right or left index finger, respectively. After value-guided choice, participants received a feedback on both options. If an option was rewarded in the current trial, the bar presenting the reward magnitude turned green (RGB: 46, 139, 60) or red (RGB: 178, 70, 70) otherwise. Every time participants were rewarded, the progress bar grew proportional to the obtained magnitude towards a goal state indicated by a golden rectangle (RGB: 184, 134, 11). The goal in the experiment was to reach the goal state as often as possible.

References

1. Wang, X.-J. Probabilistic decision making by slow reverberation in cortical circuits. *Neuron* **36**, 955–968 (2002).

2. Palminteri, S., Wyart, V. & Koechlin, E. The Importance of Falsification in Computational Cognitive Modeling. *Trends in cognitive sciences* **21**, 425–433 (2017).

3. Wilson, R.C. & Collins, A.G. Ten simple rules for the computational modeling of behavioral data. *eLife* **8** (2019).

4. Jocham, G., Hunt, L.T., Near, J. & Behrens, T.E.J. A mechanism for value-guided choice based on the excitation-inhibition balance in prefrontal cortex. *Nat. Neurosci.* **15**, 960–961 (2012).

5. Scholl, J. *et al.* A Role Beyond Learning for NMDA Receptors in Reward-Based Decision-Making—a Pharmacological Study Using d-Cycloserine. *Neuropsychopharmacology* **39**, 2900–2909 (2014).

6. Wiecki, T.V., Sofer, I. & Frank, M.J. HDDM: hierarchical bayesian estimation of the drift-diffusion model in python. *Frontiers in neuroinformatics* **7**, 14 (2013).

7. Palmer, J., Huk, A.C. & Shadlen, M.N. The effect of stimulus strength on the speed and accuracy of a perceptual decision. *Journal of vision* **5**, 1 (2005).

8. Boehm, U. *et al.* Estimating across-trial variability parameters of the Diffusion Decision Model: Expert advice and recommendations. *Journal of Mathematical Psychology* **87**, 46–75 (2018).

9. Urai, A.E., Gee, J.W. de, Tsetsos, K. & Donner, T.H. Choice history biases subsequent evidence accumulation. *eLife* **8** (2019).

10. Ratcliff, R. & Childers, R. Individual Differences and Fitting Methods for the Two-Choice Diffusion Model of Decision Making. *Decision* **2 (4)**, 237 (2015).

11. Gelman, A. & Rubin, D.B. Inference from iterative simulation using multiple sequences. *Statistical science* **7**, 457–472 (1992).

12. Fouragnan, E.F. *et al.* The macaque anterior cingulate cortex translates counterfactual choice value into actual behavioral change. *Nat. Neurosci.* **22** (2019).

13. Khalighinejad, N. *et al.* A Basal Forebrain-Cingulate Circuit in Macaques Decides It Is Time to Act. *Neuron* **105**, 370-384.e8 (2020).

14. Brockett, A.T., Tennyson, S.S., deBettencourt, C.A., Gaye, F. & Roesch, M.R. Anterior cingulate cortex is necessary for adaptation of action plans. *Proceedings of the National Academy of Sciences of the United States of America* **117**, 6196–6204 (2020).

15. Standage, D. & Paré, M. Persistent storage capability impairs decision making in a biophysical network model. *Neural networks : the official journal of the International Neural Network Society* **24**, 1062–1073 (2011).

16. Wang, X.-J. Neural dynamics and circuit mechanisms of decision-making. *Current opinion in neurobiology* **22**, 1039–1046 (2012).
