# Supplementary material for: Dissociable roles of cortical excitation-inhibition balance during patch-leaving versus value-guided decisions
Source: Nat Commun. 2021 Feb 10;12:904. doi: 10.1038/s41467-020-20875-w (PMC7875994; doi:10.1038/s41467-020-20875-w)
Supplement: Supplementary file 3 — Reporting Summary [file 41467_2020_20875_MOESM3_ESM.pdf]

nature research

Corresponding author(s): Kaiser, Luca F.

Last updated by author(s): Oct 27, 2020

Reporting Summary

Nature Research wishes to improve the reproducibility of the work that we publish. This form provides structure for consistency and transparency in reporting. For further information on Nature Research policies, see Authors & Referees and the Editorial Policy Checklist.

Statistics

For all statistical analyses, confirm that the following items are present in the figure legend, table legend, main text, or Methods section.

n/a	Confirmed	
☐	☒	The exact sample size (n) for each experimental group/condition, given as a discrete number and unit of measurement
☐	☒	A statement on whether measurements were taken from distinct samples or whether the same sample was measured repeatedly
☐	☒	The statistical test(s) used AND whether they are one- or two-sided<br>*Only common tests should be described solely by name; describe more complex techniques in the Methods section.*
☐	☒	A description of all covariates tested
☐	☒	A description of any assumptions or corrections, such as tests of normality and adjustment for multiple comparisons
☐	☒	A full description of the statistical parameters including central tendency (e.g. means) or other basic estimates (e.g. regression coefficient) AND variation (e.g. standard deviation) or associated estimates of uncertainty (e.g. confidence intervals)
☐	☒	For null hypothesis testing, the test statistic (e.g. F, t, r) with confidence intervals, effect sizes, degrees of freedom and P value noted<br>*Give P values as exact values whenever suitable.*
☐	☒	For Bayesian analysis, information on the choice of priors and Markov chain Monte Carlo settings
☐	☒	For hierarchical and complex designs, identification of the appropriate level for tests and full reporting of outcomes
☐	☒	Estimates of effect sizes (e.g. Cohen's d, Pearson's r), indicating how they were calculated

Our web collection on statistics for biologists contains articles on many of the points above.

Software and code

Policy information about availability of computer code

Data collection	Stimulus presentation was controlled by Psychtoolbox 3 (http://psychtoolbox.org/) running on Matlab R2012b (https://se.mathworks.com; The Mathworks Company, Natick, MA).
Data analysis	LCModel version 6.1.0 (Provencher, 1993) was used to analyze MRS data. SPM 12 and FieldTrip (download: 09.06.2018) running on Matlab R2018A were used to analyze MR data. Matlab R2019A was used to analyze behavioural data. We used Python 2.7.5 and the HDDM toolbox (Wiecki, Sofer & Frank (2013)) to analyze DDM data (Supplementary Materials). Our analysis was based on a script published in 2019 under a MIT license (Urai, De Gee, Tsetsos & Donner (2019); https://github.com/anne-urai/2019_Urai_choice-history-ddm/blob/master/LICENSE.md). Additionally, we have used the MEST toolbox (Version 1.6.1; Hentschke & Stüttgen ,2018: https://github.com/hhentschke/measures-of-effect-size-toolbox) to estimate measures of effect sizes.

For manuscripts utilizing custom algorithms or software that are central to the research but not yet described in published literature, software must be made available to editors/reviewers. We strongly encourage code deposition in a community repository (e.g. GitHub). See the Nature Research guidelines for submitting code & software for further information.

Data

Policy information about availability of data

All manuscripts must include a data availability statement. This statement should provide the following information, where applicable:

- Accession codes, unique identifiers, or web links for publicly available datasets
- A list of figures that have associated raw data
- A description of any restrictions on data availability

The raw MRS data that support the findings of this study are available from the corresponding author upon reasonable request. The behavioural data and a table summarising all MRS results are available under:
www.github.com/luckyluc25/ei_exp .

Field-specific reporting

Please select the one below that is the best fit for your research. If you are not sure, read the appropriate sections before making your selection.

☒ Life sciences ☐ Behavioural & social sciences ☐ Ecological, evolutionary & environmental sciences

For a reference copy of the document with all sections, see nature.com/documents/nr-reporting-summary-flat.pdf

Life sciences study design

All studies must disclose on these points even when the disclosure is negative.

Sample size

Based on our previous studies using computational models to analyse value guided choice and their relationship to neurochemistry (Jocham et al., 2012) we set the lower bound to 30 incluced datasets to reliable detect relationships.
A G*Power analysis for 90% power, alpha 0.05, based on our previous reported correlations (r=-0.598) results in a minimal sample size of 25. We recorded data of 33 participants since we expected drop outs due to insufficient quality of the MR spectra (see „Data exclusion").

Data exclusions

We excluded data of N = 4 participants from this study since at least one of their MRS voxel measurements did not pass one or more of the following quality criteria: Cramér–Rao lower bound (CRlb) < 20 %, peak width < 25 Hz full-width half-maximal (FWHM), and signal-to-noise ratio (SNRs) > 8 (thresholds were based on previous work, Dou et al., 2013). Those four participants were excluded from the behavioural analysis as well the MRS data analysis.

Replication

Our study was not designed to replicate the novel findings we report and replication with the same task would not be possible due to potential learning effects. However, parts of our findings replicate a previous publication (Jocham et al.,2012) conceptually.

Randomization

We only measured one experimental group.

Blinding

Blinding was not relevant for our study since all participants were assigned to the same experimental group. All effects were tested in a within-subject design.

Reporting for specific materials, systems and methods

We require information from authors about some types of materials, experimental systems and methods used in many studies. Here, indicate whether each material, system or method listed is relevant to your study. If you are not sure if a list item applies to your research, read the appropriate section before selecting a response.

Materials & experimental systems

n/a	Involved in the study	
☒	☐	Antibodies
☒	☐	Eukaryotic cell lines
☒	☐	Palaeontology
☒	☐	Animals and other organisms
☐	☒	Human research participants
☒	☐	Clinical data

Methods

n/a	Involved in the study	
☒	☐	ChIP-seq
☒	☐	Flow cytometry
☐	☒	MRI-based neuroimaging

Human research participants

Policy information about studies involving human research participants

Population characteristics

Thirty-three right-handed male participants [age: 26.18 ± 0.65 (mean ± SEM), range: 22-36] with normal (N=16) or corrected to normal (N=17) vision participated in this experiment. Exclusion criteria comprised a history of neurological or psychiatric illness, drug abuse and use of psychoactive drugs or medication 24 h prior to participation. Four subjects were excluded due to exceeding the threshold of valid measurements [Only metabolic measurements with a Cramér–Rao lower bound (CRlb) < 20 %, full-width half-maximal (FWHM) < 25 Hz and signal-to-noise ratio (SNRs) > 8 were included] , which was defined based on previous work (Dou et al., 2013)] . All reported results are from the remaining N = 29 subjects (mean age: 26.48 ± 0.72, range: 22-36; normal vision: N = 14; non-smoker: N = 22).

Recruitment

Participants were recruited using the participant database from the MRI Laboratory at the University Hospital Magdeburg, Germany. A possible selection bias is that participants were mostly students from the Otto-von-Guericke University Magdeburg and we only tested male participants. They are therefore not representative of the general population but of healthy, young and highly-educated males.

Ethics oversight

The study and protocols were approved by the ethics committee of the medical faculty from the Otto-von-Guericke University, Magdeburg, Germany (28/14).

Note that full information on the approval of the study protocol must also be provided in the manuscript.

Magnetic resonance imaging

Experimental design

Design type	Magnetic Resonance Spectroscopy at 7 Tesla.
Design specifications	First, a high-resolution T1 weighted scan was measured using an MPRAGE sequence for voxel placement. Afterwards, MR spectra were acquired using a stimulated echo acquisition mode (STEAM VERSE) sequence from each voxel of interest.
Behavioral performance measures	Subjects did not do any task during MR recordings.

Acquisition

Imaging type(s)	Magnetic Resonance Spectroscopy
Field strength	7
Sequence & imaging parameters	High-resolution T1 weighted scan: MPRAGE sequence (TE = 2.73 ms, TR = 2300 ms, TI = 1050 ms, flip angle = 5°, bandwidth = 150 Hz/pixel, acquisition matrix = 320 x 320 x 224, voxel size = 0.8 mm^3 isotropic).<br>MR spectra: stimulated echo acquisition mode (STEAM VERSE) sequence (128 averages, TR = 3000 ms, TE = 20 ms, mixing time = 10 ms, data size = 2048, bandwidth = 2800 Hz).
Area of acquisition	We positioned voxels in five regions of interest, including right dorsolateral prefrontal cortex (dlPFC), bilateral primary motor cortices (rM1 and lM1), perigenual anterior cingulate cortex within vmPFC (vmPFC/pgACC) and dorsal anterior cingulate cortex (dACC). The dlPFC voxel was placed on the right hemisphere within the middle frontal gyrus by using the superior frontal sulcus and the inferior frontal sulcus as anatomical landmarks. We positioned the voxel as far dorsal as possible when excluding the calvaria and all extracalvarcial structures. The average dlPFC voxel centroid across participants was estimated at MNI x = 29.79 ± 0.85, y = 37.72 ± 1.38, z = 24.21 ± 1.51 (mean ± SEM). Primary motor cortex voxels were placed on the hand knob structures, identified by their omega-like shape on the central sulcus in axial slices. Average M1 voxel centroids in standard space were estimated at MNI x = -28.97 ± 0.82, y = -18.48 ± 0.92, z = 51.86 ± 0.59 and MNI x = 31.90 ± 0.71, y = -14.76 ± 1.06, z = 49.76 ± 0.88 for rM1 and lM1, respectively. The vmPFC voxel was mediolaterally centred on the midline and dorsoventrally on the genu of the corpus callosum, with its posterior boundary just rostral to the genu. The average voxel centroid position across subjects was estimated at MNI x = -0.17 ± 0.15, y = 41.41 ± 1.29, z = 7.00 ± 0.44. The dACC voxel was placed with reference to the corpus callosum, the cingulate as well as surrounding sulci. We used the posterior border of the genu of the corpus callosum perpendicular to AC-PC orientation to centre the voxel . The average centroid voxel position across subjects was MNI x = -0.07 ± 0.19, y = 24.14 ± 0.43, z = 29.69 ± 0.47 .

Diffusion MRI ☐ Used ☒ Not used

Preprocessing

Preprocessing software	We used SPM 12 to segment data in each participants native space. Data were segmented into: grey matter, white matter, CSF, bone/skull, soft tissue and air.
Normalization	All data presented in this paper were obtained in each participants native space. Normalization was only used to obtain estimates of average voxel positions in standard space. To that end, voxel positions were interpolated onto MRI volumes using spm in Fieldtrip 20180906. Those volumes were normalized with spm12 in FieldTrip to MNI space (ft_volumenormalise). Here, we used a six-tissue type (TPM.nii) template by specifying cfg.spmmethod='new' . The centroid of the transformed voxel mask is reported as average voxel positions in MNI space.
Normalization template	Data were normalized to MNI space by using tissue probability maps (TPM.nii template from SPM 12) as implemented in ft_volumenormalise in Fieldtrip 20180906 (cfg.spmmethod='new').
Noise and artifact removal	*Describe your procedure(s) for artifact and structured noise removal, specifying motion parameters, tissue signals and physiological signals (heart rate, respiration).*
Volume censoring	*Define your software and/or method and criteria for volume censoring, and state the extent of such censoring.*

Statistical modeling & inference

Model type and settings	*Specify type (mass univariate, multivariate, RSA, predictive, etc.) and describe essential details of the model at the first and second levels (e.g. fixed, random or mixed effects; drift or auto-correlation).*
Effect(s) tested	*Define precise effect in terms of the task or stimulus conditions instead of psychological concepts and indicate whether ANOVA or factorial designs were used.*

Specify type of analysis: ☐ Whole brain ☐ ROI-based ☐ Both

Statistic type for inference (See Eklund et al. 2016)	*Specify voxel-wise or cluster-wise and report all relevant parameters for cluster-wise methods.*

Correction | *Describe the type of correction and how it is obtained for multiple comparisons (e.g. FWE, FDR, permutation or Monte Carlo).*

Models & analysis

n/a	Involved in the study
☒	☐ Functional and/or effective connectivity
☒	☐ Graph analysis
☒	☐ Multivariate modeling or predictive analysis
